# Genomic Characterization of the LEED..PEEDs, a Gene Family Unique to the *Medicago* Lineage

**DOI:** 10.1534/g3.114.011874

**Published:** 2014-08-25

**Authors:** Diana I. Trujillo, Kevin A. T. Silverstein, Nevin D. Young

**Affiliations:** Department of Plant Biology, University of Minnesota, St. Paul, Minnesota 55108

**Keywords:** secreted peptides, LEED..PEEDs, nodulation, IRLC

## Abstract

The LEED..PEED (LP) gene family in *Medicago truncatula* (A17) is composed of 13 genes coding small putatively secreted peptides with one to two conserved domains of negatively charged residues. This family is not present in the genomes of *Glycine max*, *Lotus japonicus*, or the IRLC species *Cicer arietinum*. LP genes were also not detected in a *Trifolium pratense* draft genome or *Pisum sativum* nodule transcriptome, which were sequenced *de novo* in this study, suggesting that the LP gene family arose within the past 25 million years. *M. truncatula* accession HM056 has 13 LP genes with high similarity to those in A17, whereas *M. truncatula* ssp. *tricycla* (R108) and *M. sativa* have 11 and 10 LP gene copies, respectively. In *M. truncatula* A17, 12 LP genes are located on chromosome 7 within a 93-kb window, whereas one LP gene copy is located on chromosome 4. A phylogenetic analysis of the gene family is consistent with most gene duplications occurring prior to *Medicago* speciation events, mainly through local tandem duplications and one distant duplication across chromosomes. Synteny comparisons between R108 and A17 confirm that gene order is conserved between the two subspecies, although a further duplication occurred solely in A17. In *M. truncatula* A17, all 13 LPs are exclusively transcribed in nodules and absent from other plant tissues, including roots, leaves, flowers, seeds, seed shells, and pods. The recent expansion of LP genes in *Medicago* spp. and their timing and location of expression suggest a novel function in nodulation, possibly as an aftermath of the evolution of bacteroid terminal differentiation or potentially associated with rhizobial–host specificity.

Legumes form symbiotic relationships with nitrogen-fixing soil bacteria in dedicated root organs called nodules. The establishment of this relationship is regulated through signaling processes dependent on concerted transcription reprogramming in the plant host and its symbiotic rhizobial partners (Moreau *et al.* 2011; Manoury *et al.* 2010; Karunakaran *et al.* 2009). This signaling relies on a wide range of secreted compounds such as flavonoids and secreted peptides. Secreted peptides by both the host (Wang *et al.* 2010) and by the rhizobial partners (Marie *et al.* 2003) are necessary for communication between the two organisms and successful establishment of nitrogen-fixing nodules.

There are several known secreted peptide families that are specific to legumes or have nodule-specific expression, such as the nodule-specific cysteine-rich peptides (NCRs) (Mergaert *et al.* 2003; Graham *et al.* 2004), proline-rich proteins (PRPs) (Graham *et al.* 2004), glycine-rich proteins (GRPs) (Kevei *et al.* 2002), and nodulation-related CLAVATA3 (CLV3)/ESR-related peptides (CLEs) (Mortier *et al.* 2011). Some genes, like the NCRs and a nodule-specific GRP subfamily, are composed of many members but have, so far, only been found in legumes species belonging to the inverted repeat-lacking clade (IRLC) (Kevei *et al.* 2002; Mergaert *et al.* 2003; Alunni *et al.* 2007). Mediated by NCRs with antimicrobial function (Van de Velde *et al.* 2010), most IRLC legumes host terminally differentiated bacteroids that have undergone genome endoreduplication and lost the ability to replicate. Additionally, IRLC legumes have indeterminate nodules with persistent meristems (Kereszt *et al.* 2011).

One secreted peptide family, which we call the LEED..PEEDs, was first classified as legume-specific in a study by Graham *et al.* (2004) (“group 567”) based on a series of comparative sequence homology searches between legume and nonlegume plants. The name LEED..PEED describes two conserved motifs that characterize this gene family (Figure 1), numbered according to their positional order in the *M. truncatula* A17 genome. Laporte *et al.* (2010) previously refer to the LEED..PEED family as SNARPs (small nodulin acidic RNA-binding proteins). These authors provide evidence that one member of the LEED..PEED family binds ssRNA nonspecifically; however, the term LEED-PEED indicates a more generic and appropriate description for the family as a whole, because their biological functions remain unknown. In accordance with the Mt4.0 genome annotation (www.jcvi.org/medicago/), we refer to these genes as LEED..PEEDs (abbreviated as LPs in text and Figures).

**Figure 1:**
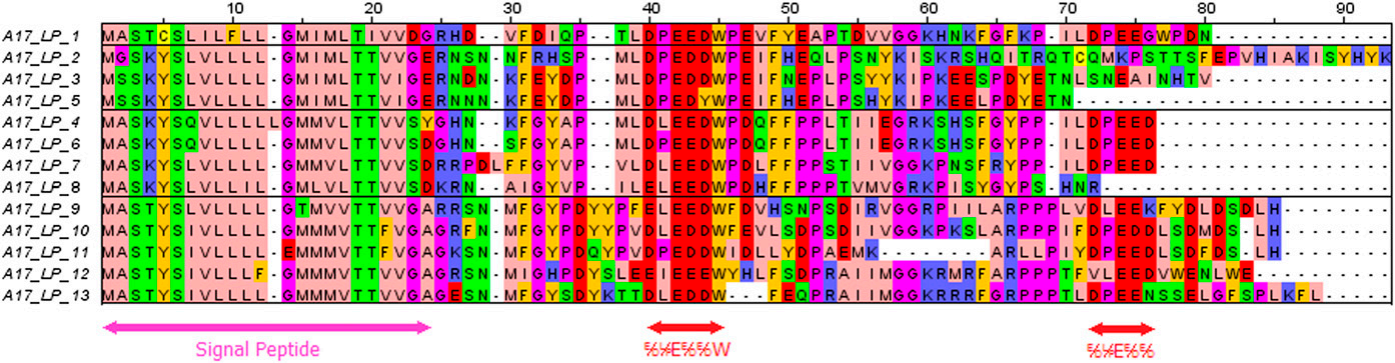
Multiple sequence alignment of A17 LP peptides. The alignment was generated by ClustalW (Larkin *et al.* 2007) and viewed with Jalview software (Waterhouse *et al.* 2009). The signal peptide sequence and conserved regions are indicated by arrows, with the consensus sequence displayed under the LEED..PEED motifs.

LP 11, described as MtSNARP2 by Laporte *et al.* (2010), is targeted to the secretory pathway in infected nodule cells. Specifically, the LP 11 signal peptide directed GFP to the membrane surrounding infection threads at 6 dpi and to the nuclear envelope at 14 dpi. Suppression of LP 11 by RNAi caused aberrant nodules with a hypertrophic outer cortex. Bacteroids in the RNAi lines initially differentiated normally but started to degenerate at 10 dpi, leading to empty peribacteroid spaces, collapse of nodule cells, and early nodule senescence. Thus, Laporte *et al.* (2010) showed that at least LP 11 is necessary for normal nodule development and function.

In the present study, the LP gene family was examined in terms of its phylogenetic, genomic, transcriptional, and sequence features. We find that this gene family, whose members are exclusively transcribed in nodules, primarily arose through tandem duplications prior to *Medicago* speciation events, but it is absent from other legumes outside of the immediate *Medicago* clade.

## Materials and Methods

### Plant genome and transcriptome sequences

Genome and transcriptome sequences for a range of legume and nonlegume species were obtained from various sources to search for the presence or absence of LPs. These sequences are summarized in Table 1.

**Table 1:** Summary of analyzed plant genomes and transcriptomes

	LPs	Genome		Transcriptome	
	Detected	Source	Version/Accession	Source	Version/Accession
*Medicago*					
A17 (*M. truncatula*)	Yes	JCVI*^a^*	Mt4.0v1	NCBI*^b^*	SRP008485
HM056 (*M. truncatula*)	Yes	NCBI*^b^*	PRJNA256006	—	—
R108 (*M. t. tricycla*)	Yes	NCBI*^b^*	PRJNA256006	—	—
HM102 (*M. sativa*)	Yes	NCBI*^b^*	PRJNA256006	LIS*^e^*	v1.0
*Trifolium pratense*	No	NCBI*^b^*	PRJNA257076	Nagy *et al.* (2013)	
*Pisum sativum*	No	—	—	NCBI*^b^*	PRJNA257308
*Cicer arietinum*	No	NCBI*^b^*	v1.0/PRJNA175619	LIS*^e^*	v2.0
*Lotus japonicus*	No	Kazusa*^c^*	Lj2.5	DFCI-GI*^f^*	Release 6.0
*Cajanus cajan*	No	NCBI*^b^*	v1.0/PRJNA72815	LIS*^e^*	v2.0
*Glycine max*	No	NCBI*^b^*	v1.1/PRJNA19861	DFCI-GI*^f^*	Release 16.0
*Phaseolus vulgaris*	No	NCBI*^b^*	v1.0/PRJNA41439	DFCI-GI*^f^*	Release 4.0
*Populus trichocarpa*	No	Phytozome*^d^*	JGI v3.0	DFCI-GI*^f^*	Release 5.0
*Arabidopsis thaliana*	No	NCBI*^b^*	TAIR10/PRJNA10719	DFCI-GI*^f^*	Release 15.0
*Oryza sativa*	No	Phytozome*^d^*	MSU release 7	DFCI-GI*^f^*	Release 19.0

a J. Craig Venter Institute (http://www.jcvi.org/medicago/).

b National Center for Biotechnology Information (http://ncbi.nlm.nih.gov/).

c Kazusa DNA Research Institute (http://www.kazusa.or.jp/lotus/).

d Phytozome v9.0 (www.phytozome.net, accessed January 2013).

e Legume Information System (comparative-legumes.org/).

f Dana Farber Cancer Institute–Gene Indices (compbio.dfci.harvard.edu/tgi/tgipage.html).

### Detection of LPs in plant genomes and transcriptomes

SPADA is a homology-based prediction program that accurately predicts small peptides at the genome level. Given a high-quality profile alignment, SPADA identifies nearly all family members with better performance than all general-purpose gene prediction programs (Zhou *et al.* 2013). SPADA was run with an e-value of 0.1 on all available plant genomes (Table 1) using an HMM profile based on the multiple sequence alignment of group 567 from Graham *et al.* (2004). Hits were then added to and used to refine the resulting multiple sequence alignments using Muscle software (Edgar 2004) for subsequent SPADA searches. SPADA searches followed by alignment refinements can be performed iteratively to find additional members of a gene family but, in the case of SPADA searches for LPs in *M. truncatula* A17, no additional genes were found after the first cycle (see *Results*).

To perform a more exhaustive search of these peptides in legumes other than *Medicago* ssp., tblastn (Altschul *et al.* 1990) searches were conducted on all the genomes and transcriptomes using *M. truncatula* A17 LPs as queries. Additionally, the Uniref90 database (http://www.ebi.ac.uk/) was scanned through an HMM search using *M. truncatula* A17 LPs. LP sequences were also used to scan the InterPro protein signature database using InterProScan at www.ebi.ac.uk/Tools/pfa/iprscan/.

### *Trifolium pratense* DNA-seq and *Pisum sativum* RNA-seq analysis

Sterilized *T. pratense* “Marathon” seeds were planted in commercial soil mix. Leaves were collected after 1 wk and frozen at −80°. DNA was extracted from frozen tissue using a Qiagen DNeasy Plant Mini Kit following manufacturer instructions. Sterilized *P. sativum* “Little Marvel” seeds were planted in sterilized Leonard jars (Leonard 1943) containing vermiculite and perlite (3:1) and immediately inoculated with *Rhizobium leguminosarum* bv. viciae USDA 2370 at a concentration of 10^8^ CFU/seed. Plants were watered with 0.25× Hoagland’s nitrogen-free nutrient solution (Hoagland and Arnon 1950) and grown in a growth chamber at 22° and with a 16-hr photoperiod. Nodules were harvested after 30 d and frozen at −80°. RNA was extracted from frozen tissue using a Qiagen RNeasy Plant Mini Kit following manufacturer instructions.

RNA and DNA samples were sent to the University of Minnesota Genomics Center for library preparation and sequencing using an Illumina HiSeq 2000. Approximately 92 million (51 bp) and 88 million (101 bp) *T. pratense* paired-end reads and 98 million (51 bp) and 96 million (101 bp) *P. sativum* paired-end reads were obtained. All reads were trimmed with Trimmomatic software (Bolger *et al.* 2014) to a minimum quality score of 20 from each end and a minimum average quality of 20 using a 4-bp sliding window. Trimmed reads smaller than 40 bp were discarded. *T. pratense* reads were assembled using ABYSS (Simpson *et al.* 2009) with default parameters and a kmer size of 33. *P. sativum* transcript assembly was conducted using Trinity software (Grabherr *et al.* 2011) with default parameters. Assembled sequences as well as raw reads were searched for A17 LP genes using SPADA as described earlier and tblastn with an e-value of 0.1.

### Transcript levels of LPs in *M. truncatula*

Illumina RNA-seq single-end reads 36 bp in size from root, nodule, seed, leaf blade, vegetative bud, and flower tissues were obtained from the Sequence Read Archive at NCBI (Accession SRP008485) (Young *et al.* 2011). RNA-seq reads were trimmed by a sliding window of 1 bp from the 3′ ends until a quality score of 20 was reached. Filtered reads were mapped to the *M. truncatula* 4.0 reference genome using Tophat (Trapnell *et al.* 2009) with maximum indel sizes of 4 bp and minimum and maximum intron lengths of 20 and 2000 bp, respectively. Cufflinks (Trapnell *et al.* 2010) was run with a maximum intron length of 2000 bp using the multi-read correct option and a reference annotation containing the 13 LPs.

### Synteny and colinearity comparisons

Regions of macro-synteny between *M. truncatula* v4.0 and *G. max* v1.1 genomes were identified and visualized using MUMmer 3 software (Kurtz *et al.* 2004) with default parameters. Gene homology patterns within these regions were analyzed using GEvo (default parameters) (Lyons and Freeling 2008) and visualized with mGSV software (Revanna *et al.* 2012), filtering out small syntenic regions (<500 bp) and joining consecutive syntenic regions within a single gene.

LPs 2–13 from *M. truncatula* ssp. *tricycla* R108 were located on a single scaffold (848), which was annotated through a best-hit blastn search using A17 annotated gene sequences. Synteny comparisons were conducted between A17 and R108 using low-resolution custom R scripts, which provided analyses similar to synteny detection with GEvo (Lyons and Freeling 2008) and visualization with mGSV (Revanna *et al.* 2012), described above. Dotplot comparisons were made between A17 and R108 or against themselves using Gepard software with default parameters (Krumsiek *et al.* 2007).

### Phylogenetic analysis of LP sequences

LP amino acid sequences of *M. truncatula* A17 and HM056 and *M. truncatula* ssp. *tricycla* R108 were aligned with ClustalW using MEGA version 5 (Tamura *et al.* 2011). The corresponding nucleotide alignment was then trimmed with removal of gapped columns, leaving 144 nucleotides to use in phylogenetic tree construction (Supporting Information, Figure S1). Independently, *M. sativa* assembled genes were aligned with the above sequences and trimmed to obtain 87 bp of aligned sequence for tree construction (Figure S2). Phylogenetic analyses were conducted using Maximum Likelihood in MEGA5 and using Bayesian Inference with MrBayes version 3.1 software (Ronquist and Huelsenbeck 2003). For both approaches, trees were inferred based on the General Time Reversible model of evolution with gamma-distributed rate variation and a proportion of invariable sites. In the Bayesian phylogenetic analysis, congruence was reached with 300,000 generations and sampling every tenth generation. Phylogenetic trees were visualized using FigTree (http://www.tree.bio.ed.ac.uk/software/figtree/). Using the Bayesian Inference trees, gene duplication histories in the A17 cluster on chromosome 7 were determined and displayed with DILTAG software (Lajoie *et al.* 2010).

## Results

### The LP gene family is specific to the *Medicago* lineage

In *M. truncatula* A17, LPs detected by SPADA ranged from 66 to 89 amino acids in length. The LPs have an average size of 75 amino acids, with a signal peptide of ∼23 amino acids. They all share a small domain of negatively charged residues (red) followed by a tryptophan residue. The C-terminal end contains another small domain of negatively charged residues in most but not all LPs (Figure 1).

LP sequences were detected in *M. truncatula* accessions A17 (reference genome) and *M. truncatula* HM056 (phylogenetically close to A17), as well as in *M. truncatula* ssp. *tricycla* (R108) and *M. sativa*. Using an e-value cutoff of 0.1, no LP hits were produced in any of the other SPADA or tblastn searches of available legume genomes or transcriptomes, including *C. arietinum*, *T. pratense*, and *P. sativum*, which are all IRLC legumes.

Only 11 LPs were initially detected in the pre-release genome of *M. truncatula* HM056, which is very closely related to A17, suggesting that the HM056 scaffolds containing LPs might be misassembled. To verify this, raw HM056 reads were aligned to the A17 LP DNA regions using Bowtie2 (Langmead and Salzburg 2012), changing default parameters to allow reads to only be mapped once. HM056 reads mapped to A17 LPs were visualized with IGV (Thorvaldsdóttir *et al.* 2012), and then HM056 LPs were manually assembled based on the visual comparison. All 13 A17 LPs were found to have HM056 LPs orthologs, generally with zero to one SNP in the coding region (Figure S2). Eleven LPs were detected in the pre-release genome of R108, and 10 were detected in *M. sativa* after manual assembly of Illumina genome reads (Figure S3).

The preliminary genome assembly of *T. pratense* was created with only ∼60× average coverage, yielding a low N50 of ∼1500 bp. To determine if this genome assembly strategy would be sensitive enough to detect LPs, *M. truncatula* ssp. *tricycla* R108 raw reads were subsampled to a level comparable with the *T. pratense* 101 bp sample, re-assembled, and searched for discovery of LP genes. For this, an initial set of 165.7 million 100-bp R108 reads was subsampled twice to obtain sets of 80 million paired-end reads. The subsamples were then assembled with ABYSS using default parameters and a kmer sweep. Assemblies were then searched for LPs using tblastn with an e-value of 0.1. With this procedure, all R108 LPs were detected in both assemblies from subsampled reads indicating that LP genes can be correctly assembled and discovered at equivalent read coverage. By contrast, tblastn searches in *T. pratense* and *P. sativum* raw reads confirmed the lack of any LP sequence homology in either species. Nonlegume genomes, transcriptomes, and the Uniref 90 database also did not contain sequences with homology to LPs. Finally, no LPs were found when searching the InterPro Database with any of the 13 *M. truncatula* A17 LP sequences.

### Genomic architecture of the LP gene family

**Figure 2:**
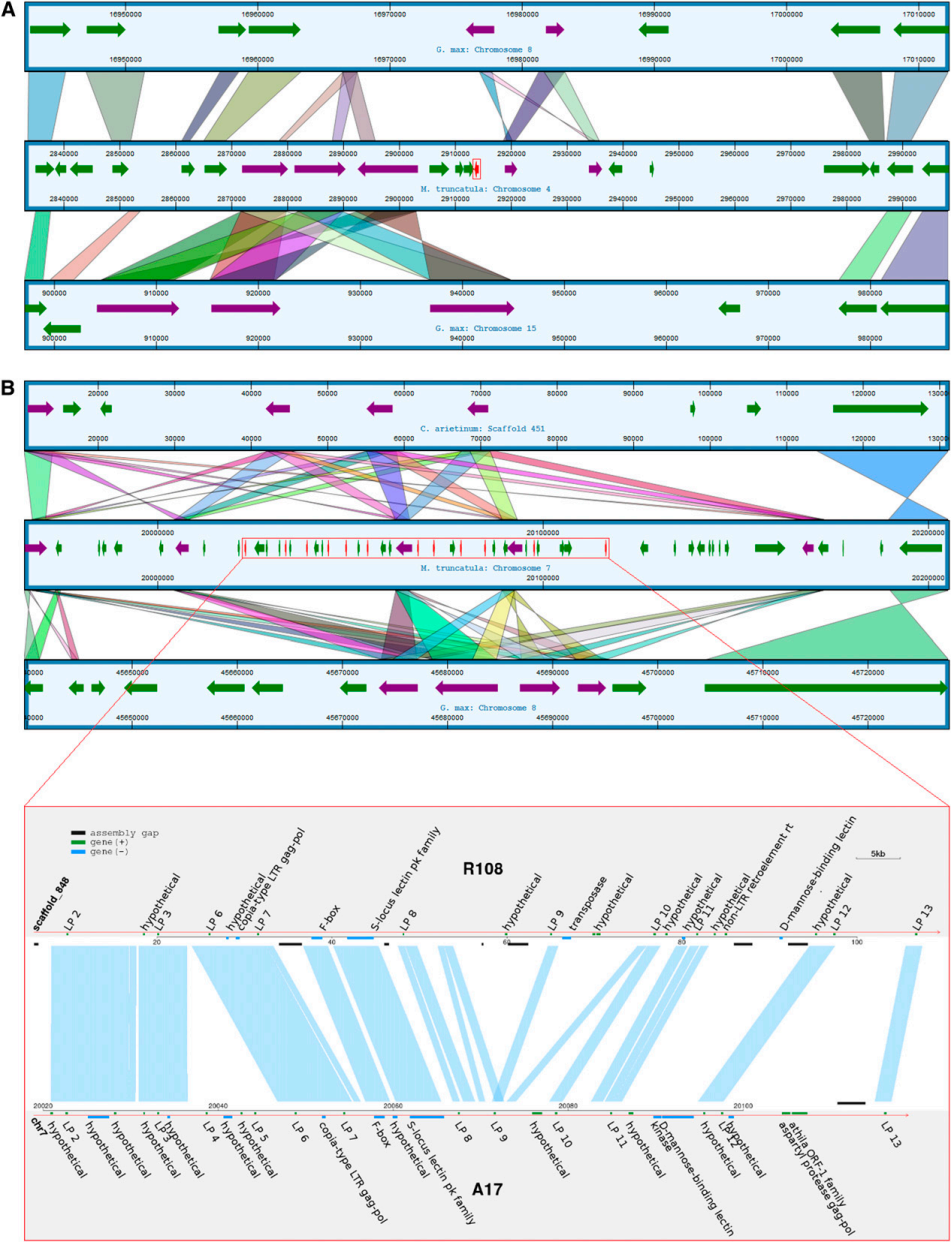
Synteny comparisons between LPs 1–13 chromosomal regions in *M. truncatula* A17 and corresponding regions in *G. max* and *C. arietinum*. Shaded bars indicate synteny between the A17 region surrounding LP 1 on chromosome 4 with *G. max* chromosomes 15 and 8 (A), and of the A17 chromosome 7 region surrounding LPs 2–13 with *G. max* chromosome 8 and *C. arietinum* scaffold 451 (B). LP genes are shown in red and the region containing them surrounded by red boxes. Neighboring genes that have also undergone tandem duplications are shown in purple, and nonduplicated neighboring genes are shown in green. (B) The ∼93-kbp LP 2–13 region of A17 chromosome 7 is magnified and compared against a ∼100-kbp region of Scaffold 848 of R108. Shaded lines between chromosomes indicate syntenic regions.

In *M. truncatula* A17, LP 1 is located on chromosome 4, whereas LPs 2–13 are located in a 93-kbp region on chromosome 7. Neighboring regions of LP 1 on A17 chromosome 4 showed synteny with *G. max* chromosomes 8 and 15 (visualized in dotplot comparisons in Figure S4a and Figure S4b, respectively). Directly neighboring the LP 1 region on A17, an analysis of corresponding syntenic regions of *G. max* shows that LP 1 in A17 (Figure 2A, red arrow) is bordered by two sets of genes on either side that have multiple copies (purple arrows). An LP ortholog is not present within either of the syntenic genomic regions in *G. max*.

Regions of synteny directly neighboring the LP 2–13 region on A17 chromosome 7 were only found on *G. max* chromosome 8 (Figure S4c), with more distantly related regions on chromosome 18 (Figure S4d). A comparison with *G. max* chromosome 8 and *C. arietinum* scaffold 451 reveals that the area has undergone numerous tandem duplications events in all three species (Figure 2B). Thus, all three species have four to five copies of a flanking gene belonging to the protein kinase family (purple arrows), although some copies have changed orientation during duplication. However, A17 has an additional set of duplicated genes—the 12 tandem LPs on chromosome 7 (red arrows)—completely absent from *G. max* and *C. arietinum*. In *M. truncatula*, all of these LPs are in the same orientation.

A synteny comparison between accessions A17 and R108 (Figure 2B) then revealed that long tracts of synteny are present in the LP 2–8 region, whereas in the LP 9–13 region synteny is interrupted in noncoding regions. The presence of 10 syntenic paralogous genes between R108 and A17 suggests that the expansion of the LP gene family in the region occurred largely before the *Medicago* subspecies split.

**Figure 3:**
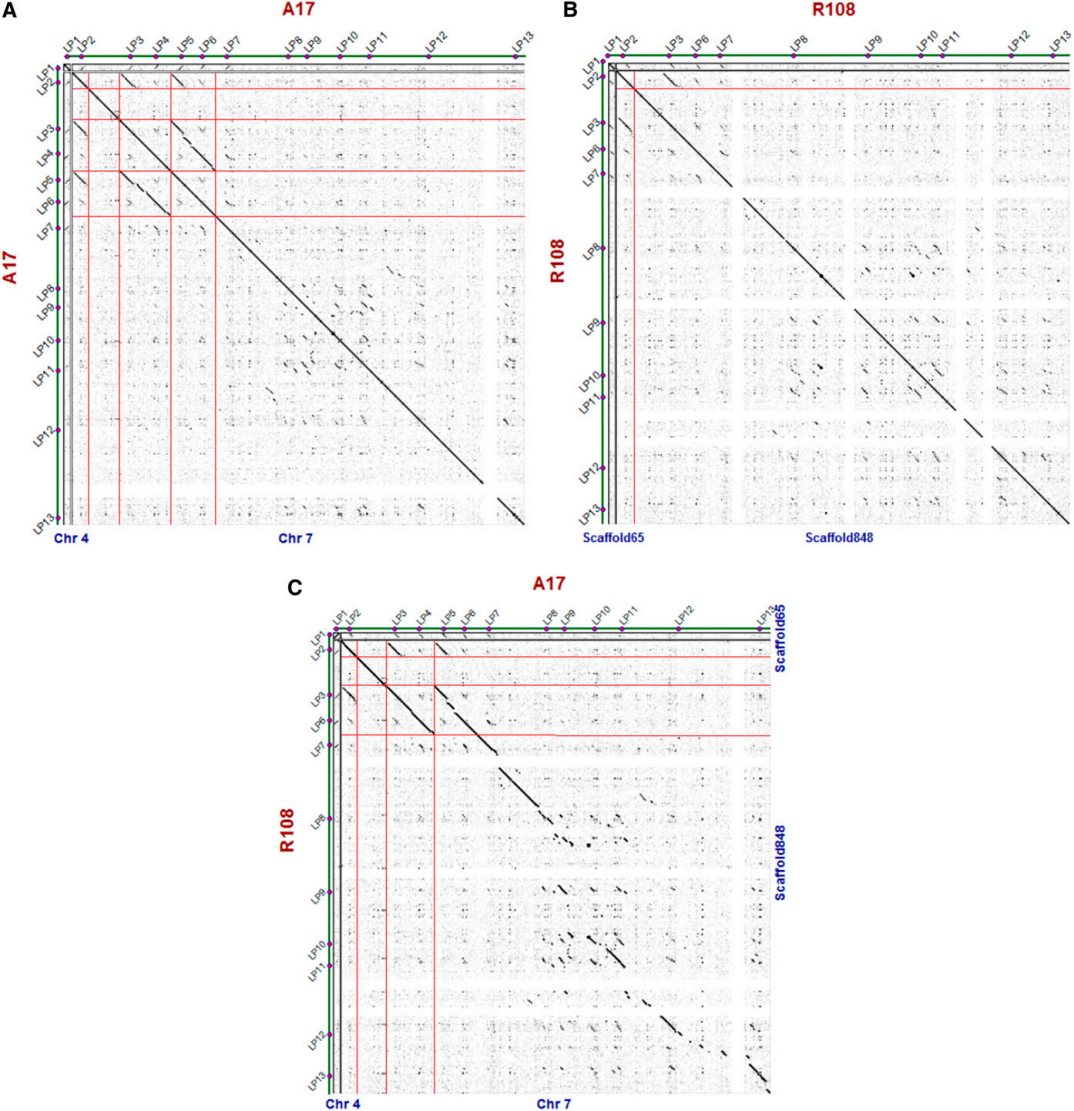
Dot plot analysis of a ∼1-kbp region in chromosome 4 and ∼100-kbp region in chromosome 7 of *M. truncatula* R108 and A17. Black diagonal lines indicate duplicated regions within A17 (A) or R108 (B) or sequence colinearity between the two organisms (C). Red horizontal and vertical lines indicate the borders of duplicated and collinear regions.

A dotplot analysis (Figure 3) shows the location of duplications in the genomic regions surrounding LPs 2–13 in A17 and R108. The duplication of a region encompassing LP 2 gave rise to LP 3 in both A17 and R108. A more recent duplication of a region encompassing LP 3 and LP 6 occurred solely in the A17 lineage, giving rise to LP 4 and LP 5 (Figure 3A), which are absent from R108 (Figure 3B). Large regions of colinearity between the two subspecies are seen surrounding LPs 2–8, with a degradation of colinearity around LPs 9–13 (Figure 3C).

### Phylogenetic relationship between LP genes

**Figure 4:**
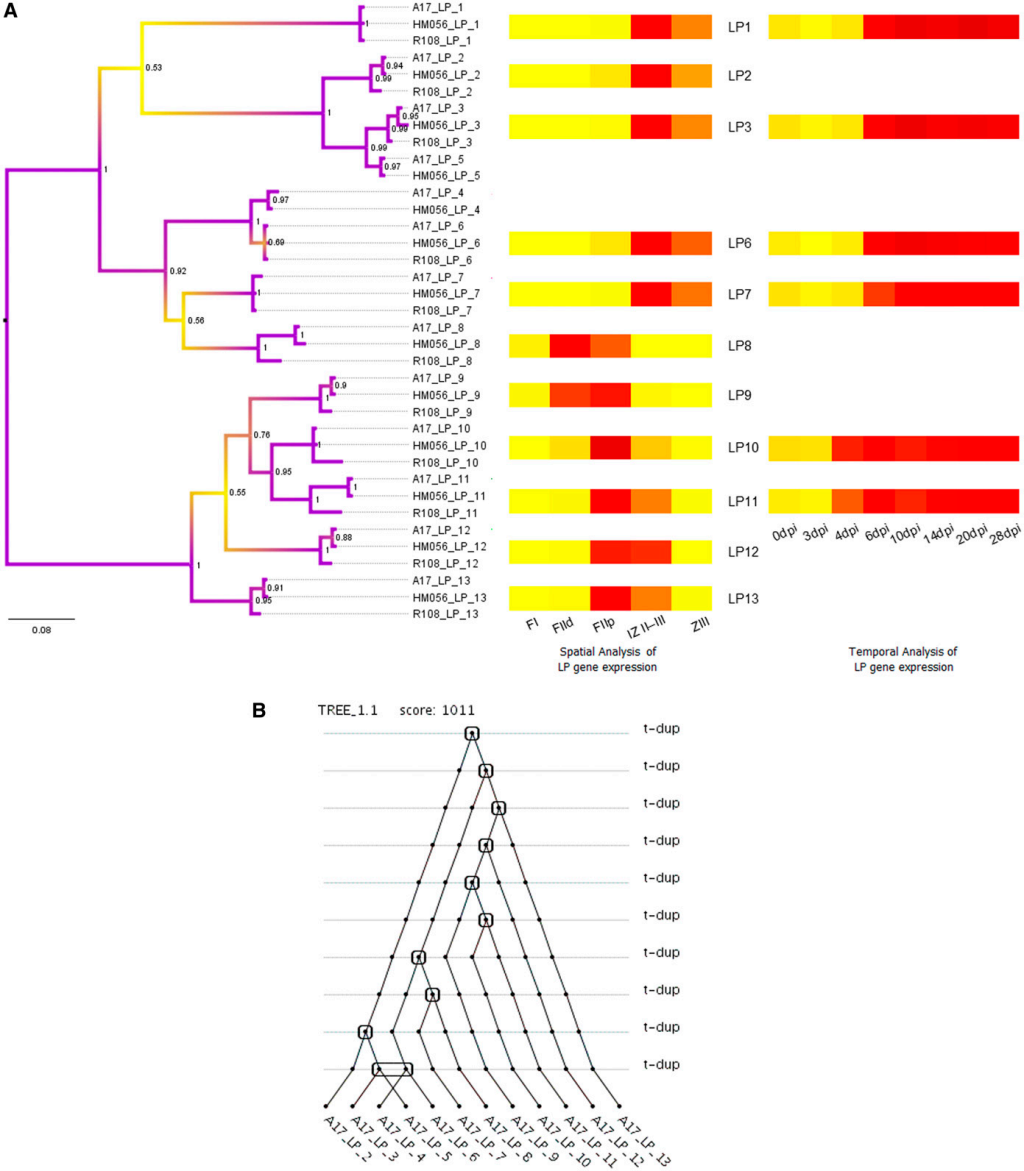
Evolutionary expansion of the LP gene family. The phylogenetic tree of A17, HM056, and R108 LP nucleotide sequences was generated through a Bayesian phylogenetic approach (A). Posterior probability values of the clades are indicated at the nodes. The heatmap insets show spatial (microdissected nodule sections) (Roux *et al.* 2014) and temporal (nodule samples taken at various time points after inoculation) (Benedito *et al.* 2008 and Carvalho *et al.* unpublished data at http://mtgea.noble.org/v3/) expression patterns for LP genes, with dark red indicating a higher transcription level for each time point or nodule section. The duplication history for A17 LP genes was inferred for the Bayesian Inference trees using DILTAG software (B). Rounded squares indicate duplication events, whereas the rounded rectangle indicates a double duplication.

To determine the evolutionary relatedness of the LPs, an unrooted phylogenetic tree was constructed based on an alignment of their nucleotide sequences (Figure S1) using Bayesian Inference and Maximum Likelihood approaches. Inferred relationships between genes were largely similar using the two approaches, although the relationship of LP 7 and LP 8 was unresolved using the Maximum Likelihood approach (data not shown). Figure 4A shows the tree inferred for A17, HM056, and R108 using Bayesian Inference, with posterior probabilities at the nodes. Including *M. sativa* in the analysis resulted in fewer resolved nodes (Figure S5), probably due to the incomplete LP gene sequence information for this species (Figure S3).

Relationships among LP orthologs reflect the phylogenetic relatedness between the accessions (Yoder *et al.* 2013). As expected, A17 and HM056 sequences tend to cluster together due to a closer phylogenetic relationship between the two accessions, whereas *M. truncatula* ssp. *tricycla* and *M. sativa* sequences are less closely related (Figure S5). The tree topology shows an order of relatedness for LPs 2–13, which is in concordance with tandem gene duplications, most of which took place before *Medicago* speciation events. Another duplication appears to have occurred after the *M. sativa* speciation event, giving rise to LP 8 in *M. truncatula* (Figure S5). An additional duplication occurred solely in the *M. truncatula* A17 and HM056 lineage in a genomic region encompassing two genes, giving rise to LPs 3–6 (Figure 4B), as previously indicated by the dotplot analysis (Figure 3A).

### The LEED..PEED family is nodule-specific in *M. truncatula*

LP genes 1–3 and 5, 4 and 6–8, and 9–13 cluster into separate groups (Figure 4A), which led us to investigate whether genes belonging to the different clusters showed a difference in timing of LP nodule expression. Expression levels for LPs 10 and 11 (Figure 4A, inset) (http://mtgea.noble.org/v3/ and Roux *et al.* 2014) become noticeable early during nodulation by 4 dpi and peak at 6 dpi (shown as dark red bars in the heatmap), whereas LPs 1, 3, 6, and 7 had higher expression levels that peaked at 10 dpi and were maintained through 20 dpi. On a spatial scale, a clear difference in expression trends among nodules sections was seen between sets of genes. LP 8 and LP 9, which belong to different phylogenetic clusters, both showed enriched expression in the distal (FIId) and proximal (FIIp) fractions of nodule zone II, which contains bacterial and nodule cells undergoing infection (FIId) and differentiation (FIIp). LPs 10–13 were more highly expressed in FIIp, which contains rhizobia undergoing endoreduplication, and in interzone II–III (IZ II–III). LPs 1–3, 6, and 7 had higher transcript levels in IZ II–III and the nitrogen fixation zone (ZIII), which contains fully differentiated bacteroids. None of the LP genes was expressed in the nodule meristem (FI). An analysis of transcript levels across several tissues showed that all 13 LPs are transcribed in *M. truncatula* A17 nodules at high levels, with little to no expression in roots or other tissues (Table 2).

**Table 2:** Transcript abundance (FPKM) of LP genes in six *M. truncatula* A17 tissues

Gene	Root 4 wk	Nodule	Seed Pod	Blade 4wk	Bud 4 wk	Open Flower
LP 1	2	6120	0	0	0	0
LP 2	0	100	0	0	0	0
LP 3	3	1922	0	0	0	0
LP 4	0	623	0	0	0	0
LP 5	5	8805	0	0	0	0
LP 6	0	973	0	0	0	0
LP 7	2	1593	0	0	0	0
LP 8	0	104	0	0	0	0
LP 9	0	113	0	0	0	0
LP 10	6	396	2	0	0	0
LP 11	0	842	0	0	0	0
LP 12	0	102	0	0	0	0
LP 13	6	2087	0	0	0	0

FPKM values from RNA-seq expression analysis.

## Discussion

Like the NCRs and GRPs, the LEED..PEED gene family is also specific to IRLC legumes. In the case of LPs, however, this lineage-specific expansion is found in a much narrower range of species. Members of the IRLC group form indeterminate nodules with persistent meristems, whereas a subset of these legumes, including *Medicago* ssp., host terminally differentiated bacteroids (Mergaert *et al.* 2006). Chickpea is an IRLC legume with indeterminate nodules, although rhizobia do not terminally differentiate (Oono *et al.* 2010). SPADA and tblastn searches of the available *C. arietinum* genome, which has an estimated 90.8% gene coverage (Varshney *et al.* 2013), suggested that LPs are absent in this species. Additionally, synteny analysis of the scaffold syntenic to the LP 2–13 region shows a clear absence of LP genes in *C. arietinum* (Figure 2). Furthermore, LPs were not detected in *Trifolium* and *Pisum*, which are even more closely related to *Medicago* and also host terminally differentiated rhizobia. The absence of LP genes in *Cicer*, *Trifolium*, and *Pisum* suggests that these proteins are not essential determinants of indeterminate nodule formation or bacteroid terminal differentiation, although they may have arisen as a consequence of these traits. Given that *Trifolium* and *Pisum* species are both nodulated by *Rhizobium leguminosarum* and that *Medicago* species are nodulated by *Sinorhizobium meliloti*, the biological function of LP genes could be related to *Medicago* species’ interaction with its rhizobial partner. *Melilotus* and *Trigonella*, sister genera to *Medicago* in the Trigonellinae (Steele *et al.* 2010), also associate with *S. meliloti* (Roumiantseva *et al.* 2002), so it will be interesting to determine whether these legumes have LP genes (preliminary data indicate LP genes may be found within *Melilotus*, unpublished data).

The LP genes were considered to be of particular interest because all 13 *M. truncatula* A17 genes had very high expression in nodules compared with other tissues, based on RNA-seq data from Young *et al.* (2011) (Table 2). Phylogenetic trees of the LPs show that genes 1–3 and 5, 4 and 6–8, and 9–13 of *M. truncatula* A17 cluster separately, suggesting possible functional differences between the groups. In our analysis of nodulation time-series expression data available at http://mtgea.noble.org/v3/, LP 10 and LP 11 transcription began and peaked earlier than that of LPs 1, 3, 4, and 7. Other studies have shown that LPs 1 and 13 belong to separate expression patterns, being activated in mature and immature nodules, respectively (Manoury *et al.* 2010), whereas LP 11 is directed to membranes surrounding infection threads (Laporte *et al.* 2011). LP genes 8–13 had higher expression in the infection zone with cells undergoing differentiation. Thus, the gene cluster containing LPs 9–13 as well as LP 8 may have an earlier role in nodulation. Potentially, LPs in this cluster might be necessary in the maintenance of functional bacteroids as they undergo differentiation, as suggested by an aberrant nodulation phenotype after LP 11 was suppressed (Laporte *et al.* 2010). Notably, an observable phenotype after suppression of just a single LP gene suggests that these genes may have distinct, nonredundant functions.

The LP gene family arose after the *Pisum-Medicago* split, which has been estimated to have occurred ∼25 mya (Lavin *et al.* 2005). It appears the LP gene family evolved by tandem duplication within this time frame. The comparatively rapid expansion and subsequent fixation of multiple LP copies suggest that higher LP copy numbers have provided a selective advantage to *Medicago* plants. The two most recent duplications occurred less than 7 mya, one after the *M. sativa* speciation event (time estimate based on *matK* substitution rates) (Lavin *et al.* 2005) and one after the much more recent A17–R108 split.

Possible theories about how the LP gene family emerged include *de novo* evolution, domain shuffling, and horizontal gene transfer (HGT). None of these theories can be ruled out. *De novo* families that have undergone lineage-specific expansion typically have structurally simple domains such as α-helices or histidine/cysteine-rich regions that stabilize molecules (Lespinet *et al.* 2002). Although the LPs lack such features, their small size may counteract the need for strict protein stabilization. At this point, there is no evidence for HGT from another species. Codon usage patterns, which can be used to distinguish native genes within a species from foreign genes, were calculated for the mature peptide region of LP genes using CAIcal software (Puigbò *et al.* 2008). These values did not stray from the average of all *M. truncatula* genes (data not shown). However, the short length of these genes and/or the amount of time since emergence may make it impossible to rule out the possibility of HGT through a codon usage index analysis alone. Evidence for domain shuffling was also not found. Although the LPs could have acquired their signal peptide from another region in the genome, the mature peptide region of LP genes does not have homology to any other gene within *Medicago* or its immediate *Trifolium* and *Pisum* relatives.

Lineage-specific expanded gene families generally have roles in an organism’s response to stress or pathogens, either as structural components or as mediators of specificity within signaling pathways (Lespinet *et al.* 2002). Gene duplication provides new material on which selection can act without harming the original function of the gene. A selection pressure that favors high gene copy numbers is often associated with positive *d_N_*/*d_S_* ratios (in which amino acid changes are advantageous), due to an expansion event followed by diversification for specificity-related roles. However, an analysis of *d_N_*/*d_S_* ratios revealed that the LP gene family as a whole tends toward purifying selection (data not shown), with amino acid changes being deleterious. Perhaps amplification of this family was driven by the genomic region rather than a biological need for a diverse set of LPs. Whatever the driving force behind increasing copy numbers, the LPs are a lineage-specific innovation that has been directed toward a function in nodulation.

The LP gene family has undergone recent expansion, mediated through one distant and several rounds of local tandem duplication events. Likewise, most GRPs and NCRs in *M. truncatula* are found in local clusters, generally facing the same orientation (Alunni *et al.* 2007). LPs differ from NCRs and GRPs in that there are fewer peptides in this gene family with comparatively lower variation in copy number across *Medicago* species. Another difference is the much narrower range of legume species in which LPs are found. The lack of sequence similarity of LPs with genes in any other legume plant, including *Pisum* and *Trifolium*, suggests that this gene family emerged *de novo* (although its origins remain unclear), expanded rapidly, and became fixed in relatively short evolutionary time. Additionally, it appears to be specifically directed toward nodulation or rhizobial interactions.

This study validates the use of comparative bioinformatic approaches toward identifying genes of potential biological interest. Future studies should focus on the different biological roles of the LP members and determining whether these proteins are present in any other legume species that are closely related to *Medicago*, such as *Melilotus* and *Trigonella* species.

## Supplementary Material

Supporting Information
