# Supplementary material for: Genomic Characterization of the LEED..PEEDs, a Gene Family Unique to the Medicago Lineage
Source: G3 (Bethesda). 2014 Aug 25;4(10):2003–12. doi: 10.1534/g3.114.011874 (PMC4199706; doi:10.1534/g3.114.011874)
Supplement: Supporting Information [file supp_4_10_2003__index.html]

Genomic Characterization of the LEED..PEEDs, a Gene Family Unique to the Medicago Lineage — Supporting Information 

# Genomic Characterization of the LEED..PEEDs, a Gene Family Unique to the *Medicago* Lineage

## Supporting Information for Trujillo, Silverstein, and Young, 2014

**Files in this Data Supplement:**

- Supporting Information - Figures S1-S5 (PDF, 1 MB)
- Figure S1 - Aligned LP DNA sequences from A17, HM056 and R108. (PDF, 385 KB)
- Figure S2 - Aligned LP DNA sequences from A17, HM056, R108 and *M. sativa*. (PDF, 798 KB)
- Figure S3 - Multiple sequence alignment of LPs from *M. truncatula* accessions A17 and HM056, R108 and *M. sativa*. (PDF, 593 KB)
- Figure S4 - Dotplot comparisons between *M. truncatula* and *G. max* in regions surrounding *M. truncatula* LPs. (PDF, 457 KB)
- Figure S5 - Phylogenetic tree of A17, HM056, R108 and *M. sativa* LP nucleotide sequences. (PDF, 350 KB)
