# Supplementary material for: Genomic Characterization of the LEED..PEEDs, a Gene Family Unique to the Medicago Lineage
Source: G3 (Bethesda). 2014 Aug 25;4(10):2003–12. doi: 10.1534/g3.114.011874 (PMC4199706; doi:10.1534/g3.114.011874)
Supplement: Supporting Information [file supp_g3.114.011874_011874SI.pdf]

G3·Genes | Genomes | Genetics

Genomic characterization of the LEED..PEEDs, a gene family unique to the *Medicago* lineage

Diana I. Trujillo [*], Kevin A. T. Silverstein [*], Nevin D. Young [*]

[*]Department of Plant Biology, University of Minnesota, Saint Paul, MN 55108

All raw sequence read data have been deposited at NCBI (http:// ncbi.nlm.nih.gov/) under BioProject accession numbers PRJNA257076, PRJNA257308, and PRJNA256006.

Corresponding author:
Nevin Young
495 Borlaug Hall, 1991 Upper Buford Circle, University of Minnesota
St. Paul MN 55108
Phone: 612-625-2225
Email: neviny@umn.edu

DOI: 10.1534/g3.114.011874

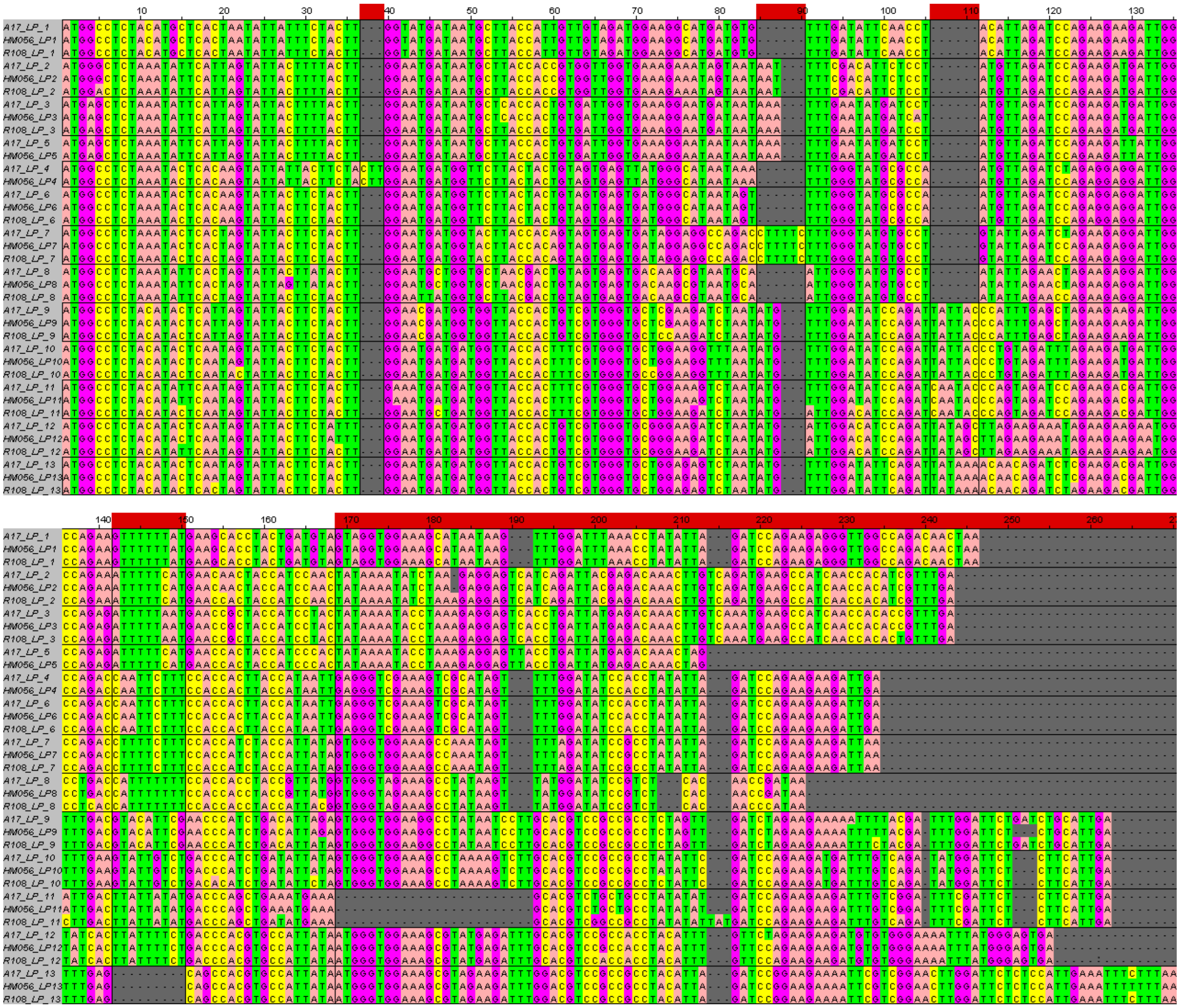

Figure S1 Aligned LP DNA sequences from A17, HM056 and R108. Columns that were omitted for the phylogenetic analysis are highlighted with red headers. Phylogenetic trees were based on the remaining 144 nucleotides.

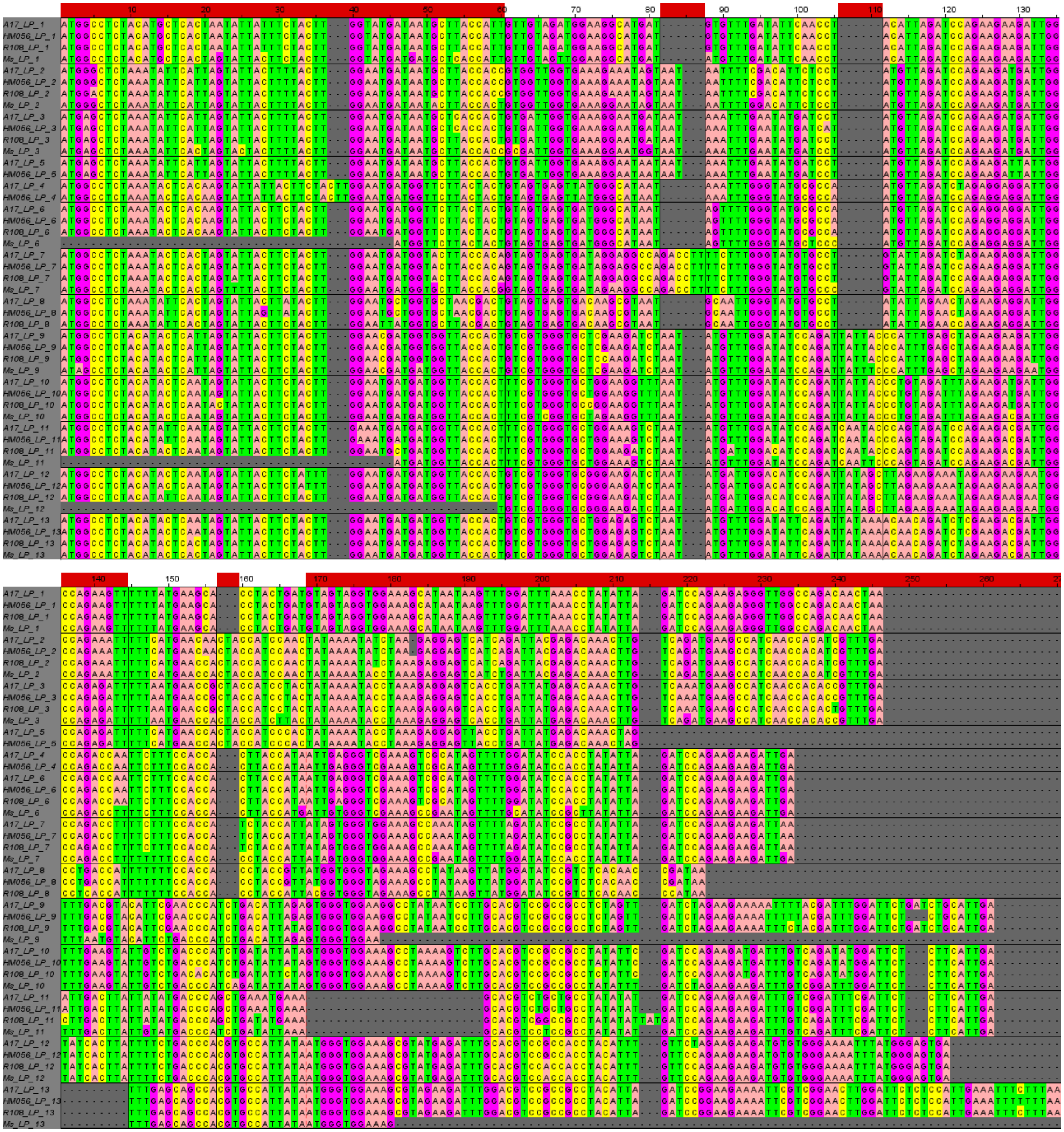

Figure S2 Aligned LP DNA sequences from A17, HM056, R108 and *M. sativa*. Columns that were omitted for the phylogenetic analysis are highlighted with red headers. Phylogenetic trees were based on the remaining 87 nucleotides.

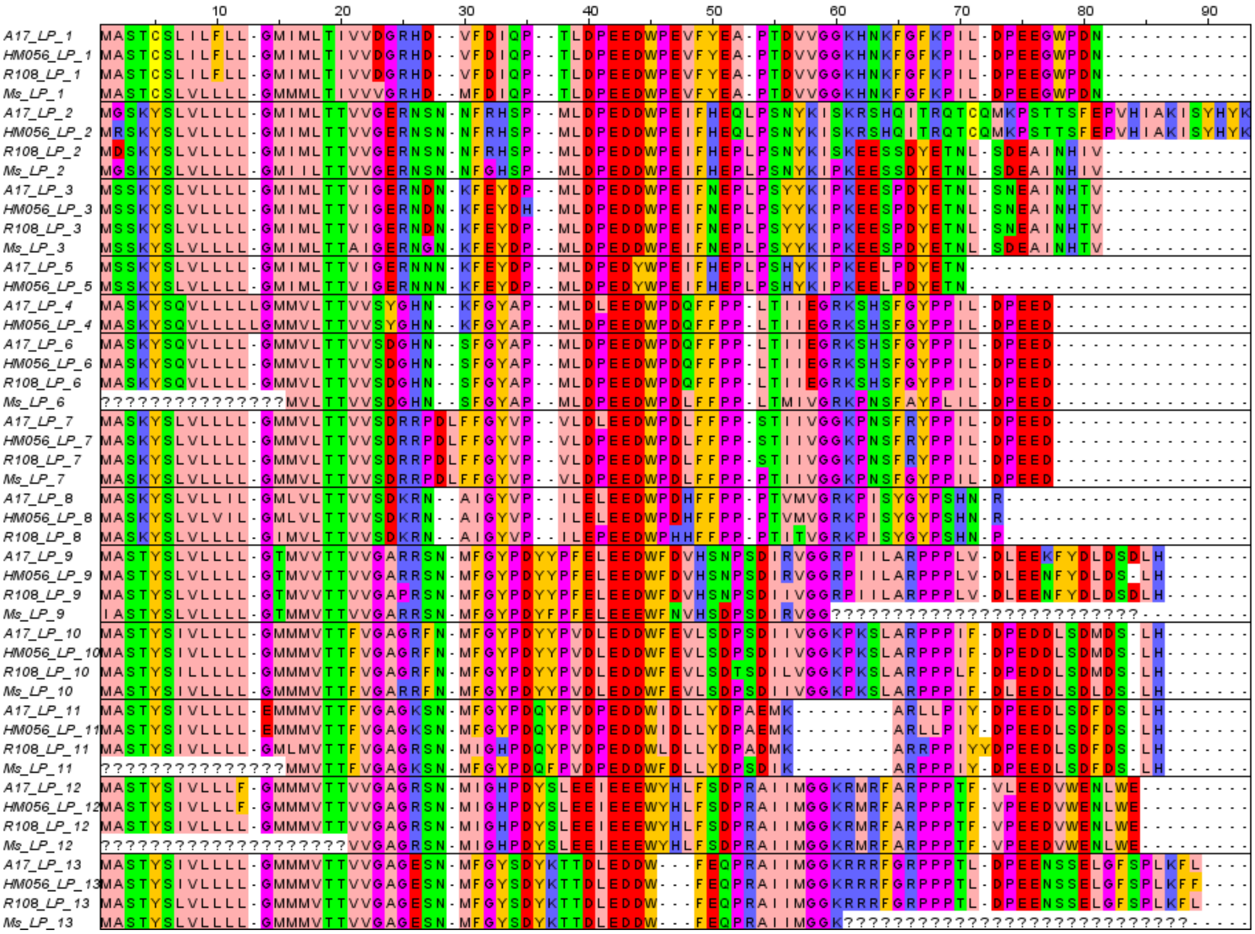

Figure S3 Multiple sequence alignment of LPs from *M. truncatula* accessions A17 and HM056, R108 and *M. sativa*. Unknown *M. sativa* residues are indicated with "?".

A.

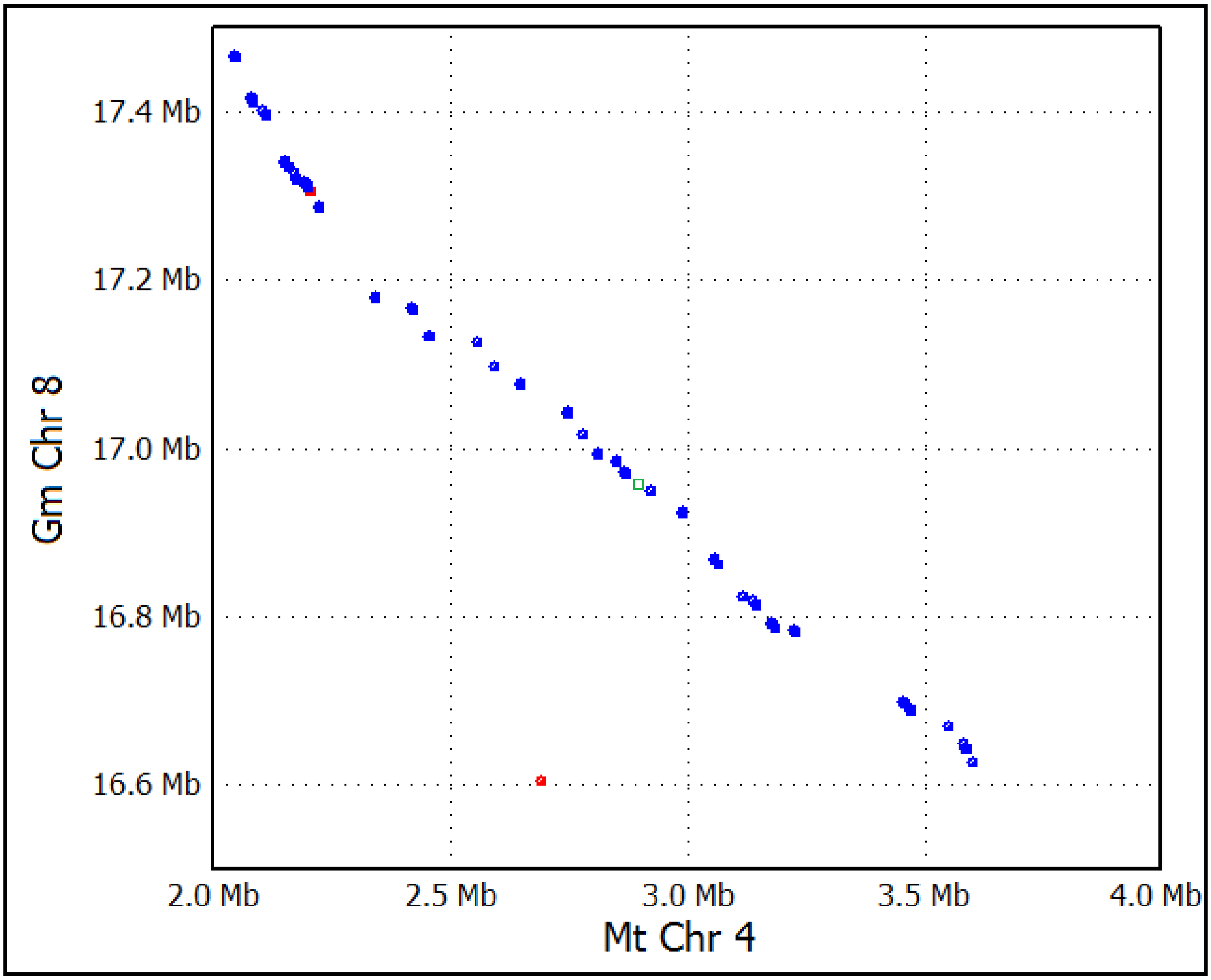
17.4 Mb
17.2 Mb
17.0 Mb
16.8 Mb
16.6 Mb
Gm Chr 8
2.0 Mb
2.5 Mb
3.0 Mb
3.5 Mb
4.0 Mb
Mt Chr 4

B.

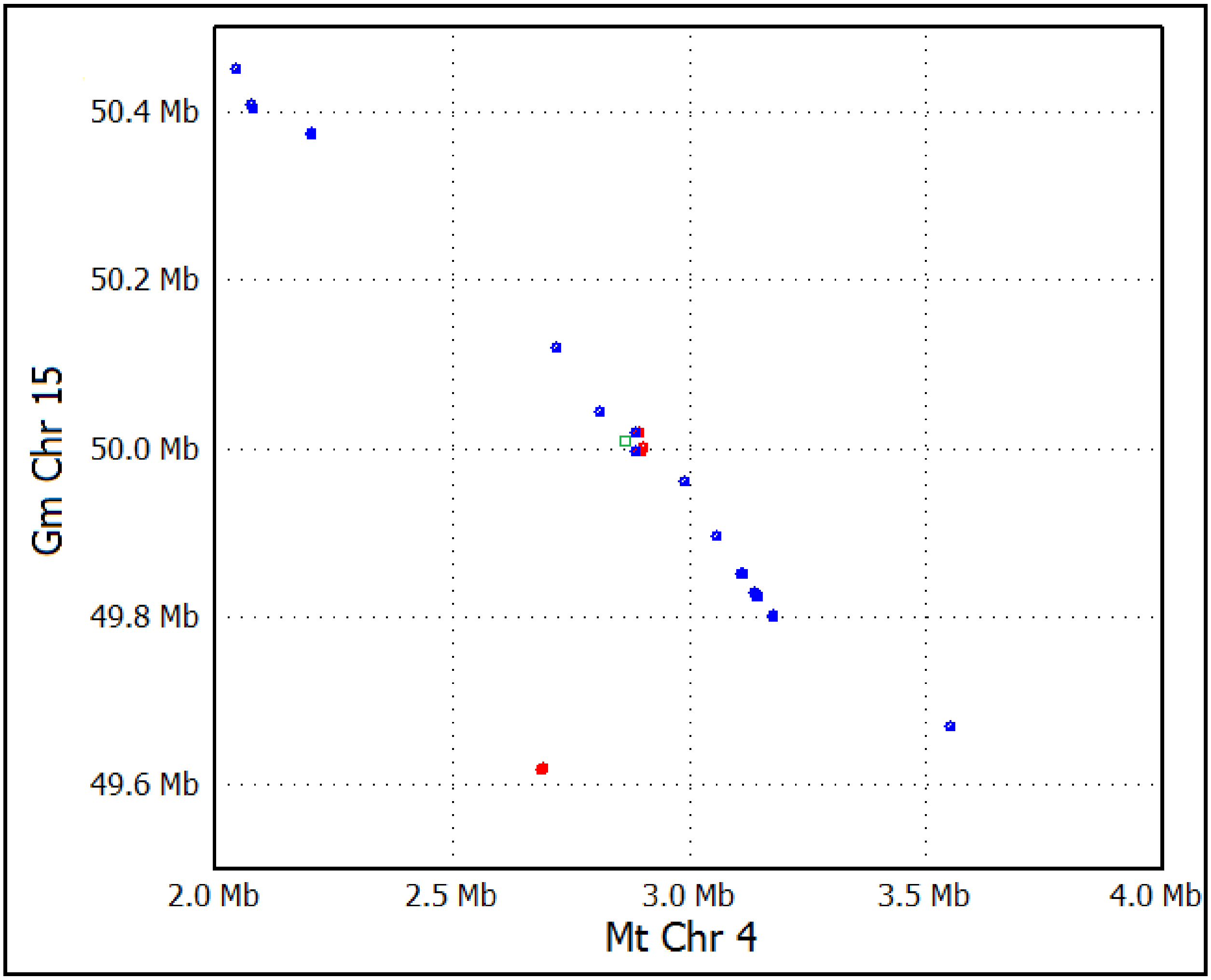
50.4 Mb
50.2 Mb
50.0 Mb
49.8 Mb
49.6 Mb
Gm Chr 15
2.0 Mb
2.5 Mb
3.0 Mb
3.5 Mb
4.0 Mb
Mt Chr 4

C.

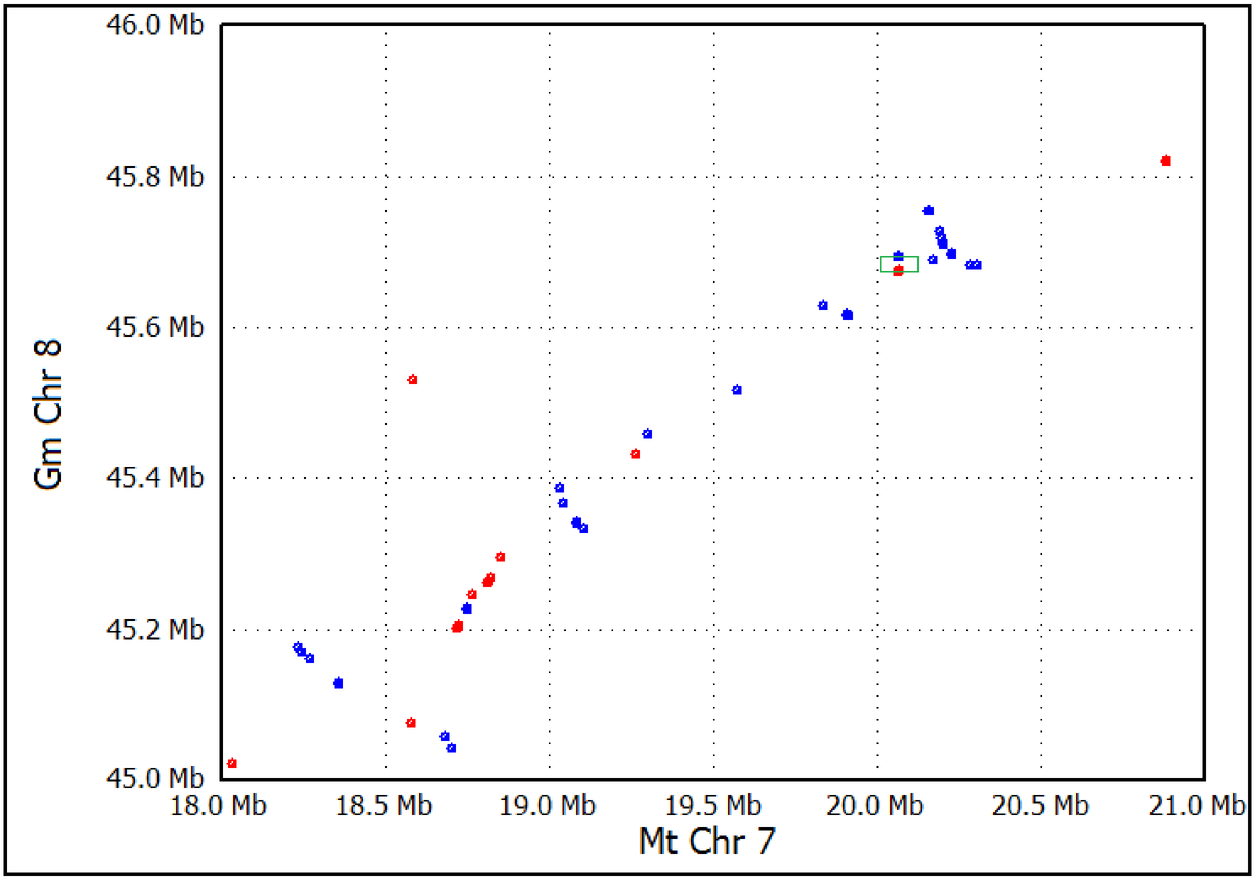
46.0 Mb
45.8 Mb
45.6 Mb
45.4 Mb
45.2 Mb
45.0 Mb
Gm Chr 8
18.0 Mb
18.5 Mb
19.0 Mb
19.5 Mb
20.0 Mb
20.5 Mb
21.0 Mb
Mt Chr 7

D.

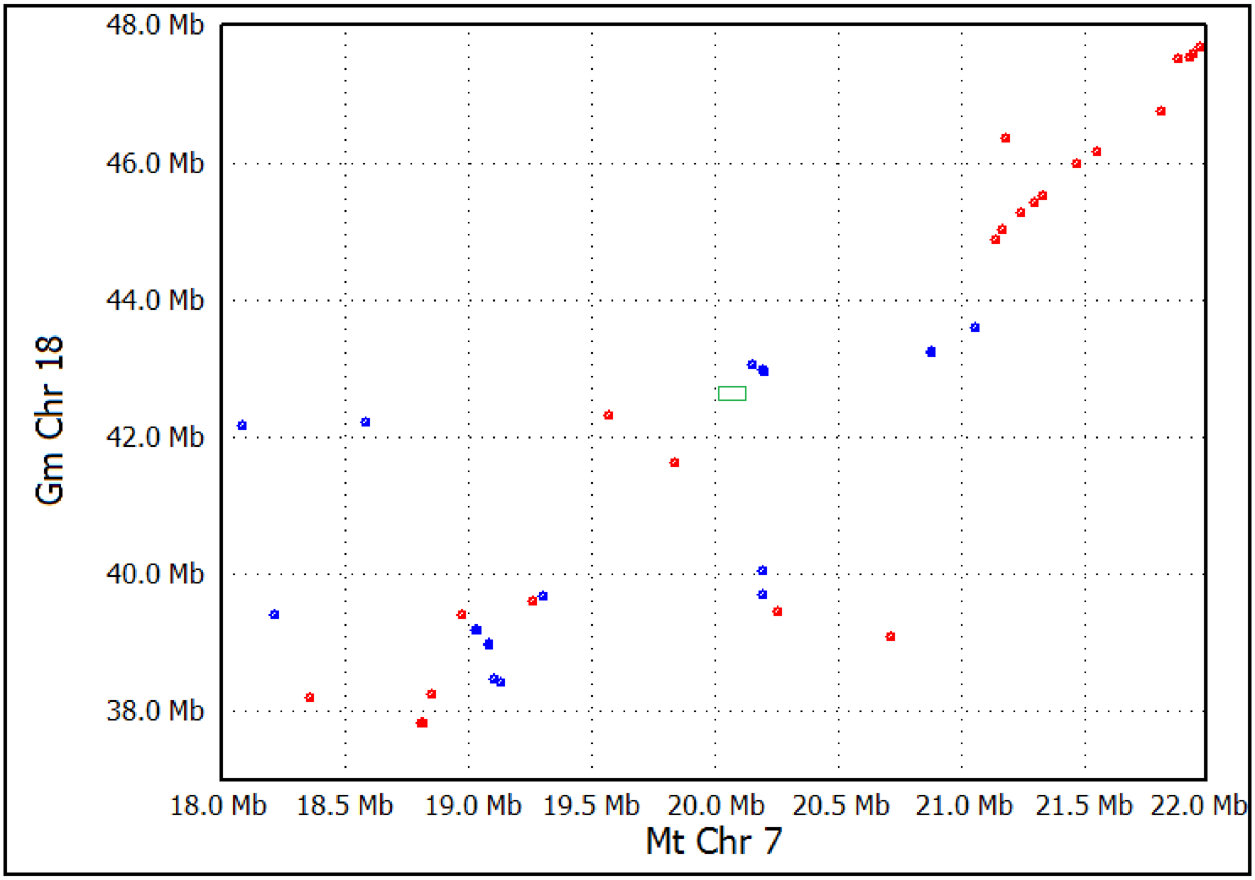

Figure S4 Dotplot comparisons between *M. truncatula* and *G. max* in regions surrounding *M. truncatula* LPs. Panels show syntenic regions of *G. max* chromosomes 8 a) and 15 b) compared against a 2 Mb region in A17 chromosome 4, corresponding to 0.5% of the total *M. truncatula* genome. Syntenic regions of *G. max* chromosomes 8 c) and 18 d) are compared against a 3-4 Mb region of A17 chromosome 7. Red dots indicate regions of sequence similarity with a forward orientation, while blue dots indicate reverse matches. The LP region in *M. truncatula* is shown with green rectangles.

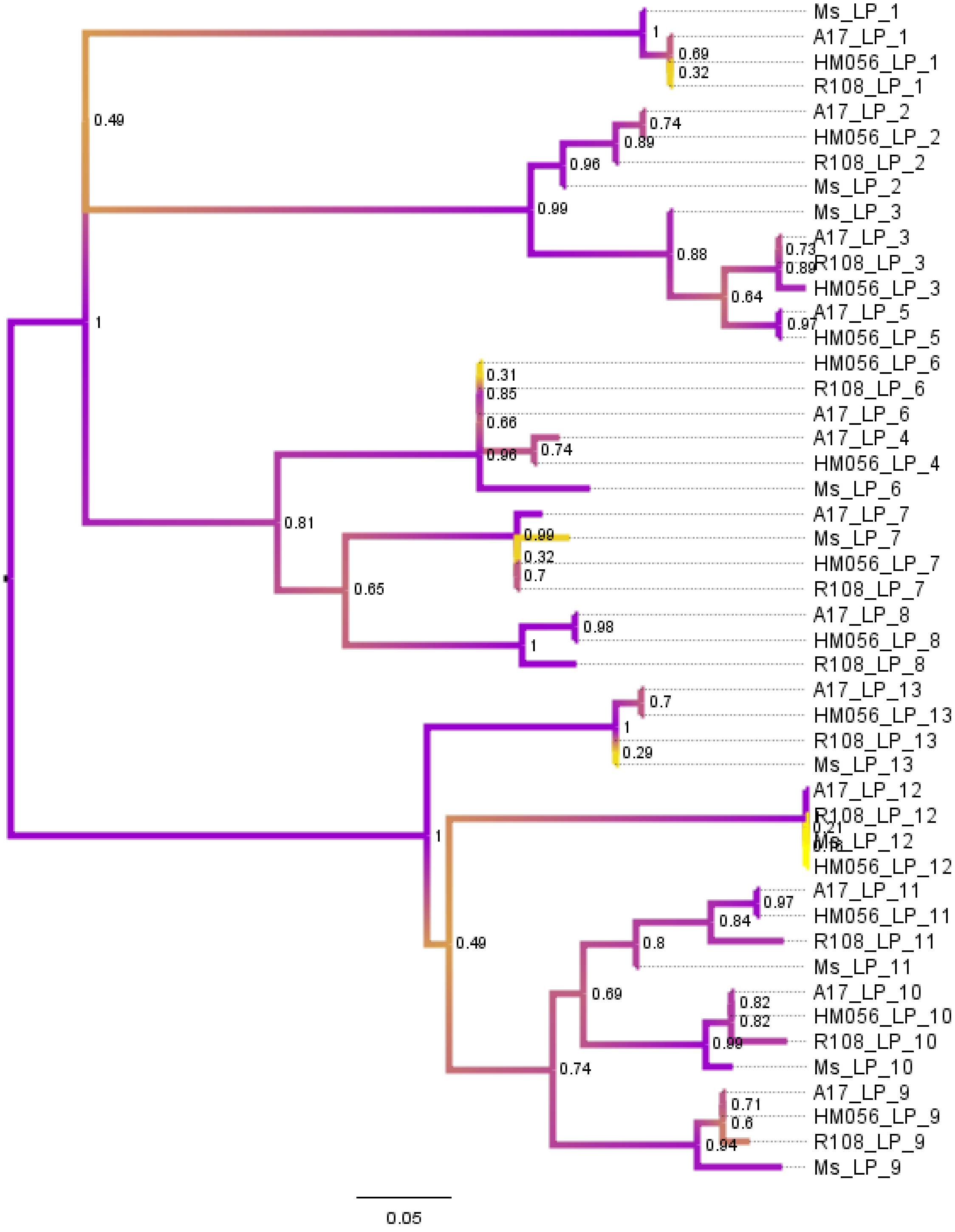

Figure S5 Phylogenetic tree of A17, HM056, R108 and *M. sativa* LP nucleotide sequences. The tree was generated through Bayesian Inference and visualized with FigTree software. Posterior probability values of the clades are indicated at the nodes.
