# Supplementary material for: Genomic Characterization of the LEED..PEEDs, a Gene Family Unique to the Medicago Lineage
Source: G3 (Bethesda). 2014 Aug 25;4(10):2003–12. doi: 10.1534/g3.114.011874 (PMC4199706; doi:10.1534/g3.114.011874)
Supplement: Supporting Information [file supp_g3.114.011874_FigureS1.pdf]

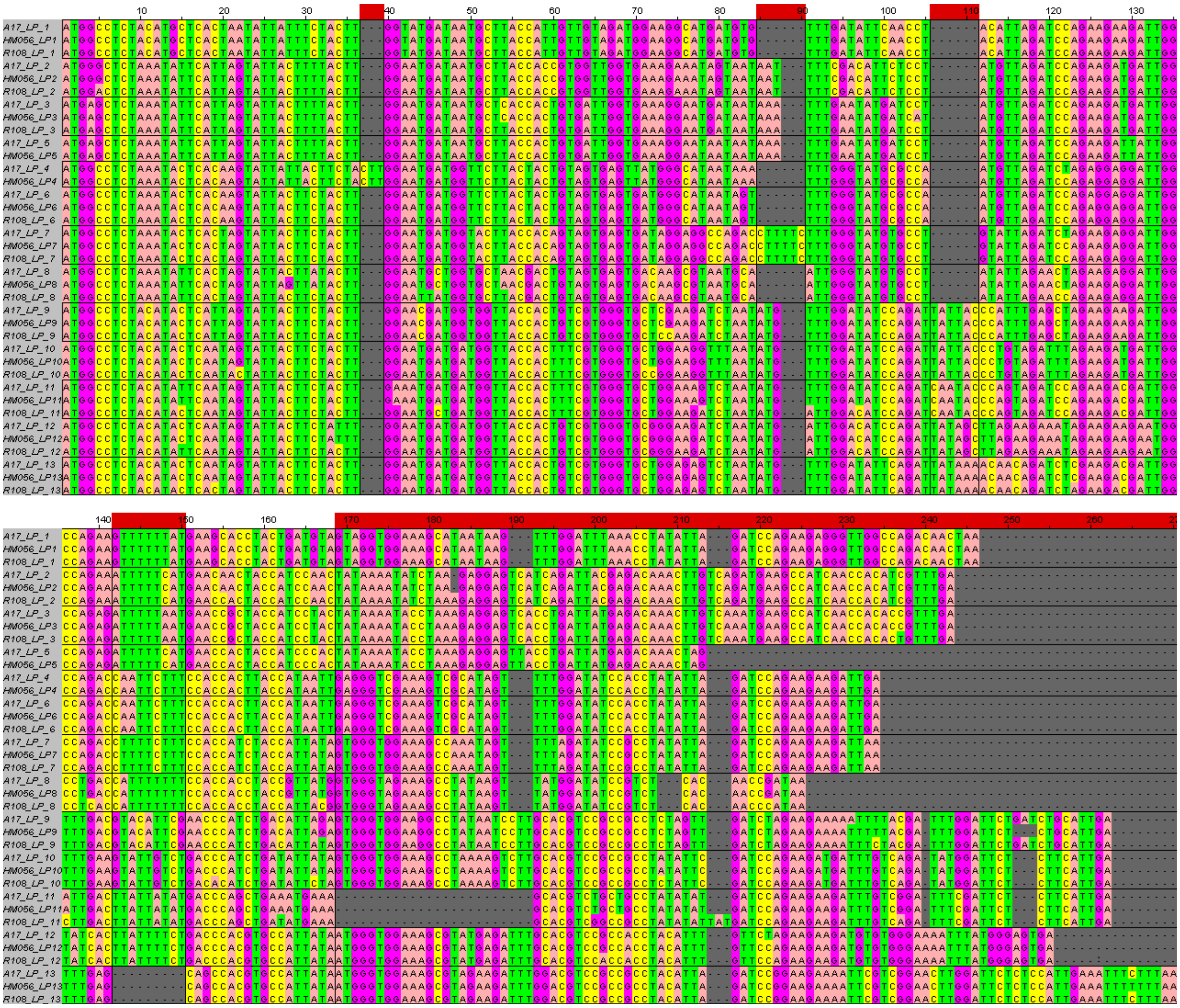

**Figure S1 Aligned LP DNA sequences from A17, HM056 and R108.** Columns that were omitted for the phylogenetic analysis are highlighted with red headers. Phylogenetic trees were based on the remaining 144 nucleotides.
