# Supplementary material for: Genomic Characterization of the LEED..PEEDs, a Gene Family Unique to the Medicago Lineage
Source: G3 (Bethesda). 2014 Aug 25;4(10):2003–12. doi: 10.1534/g3.114.011874 (PMC4199706; doi:10.1534/g3.114.011874)
Supplement: Supporting Information [file supp_g3.114.011874_FigureS4.pdf]

A.

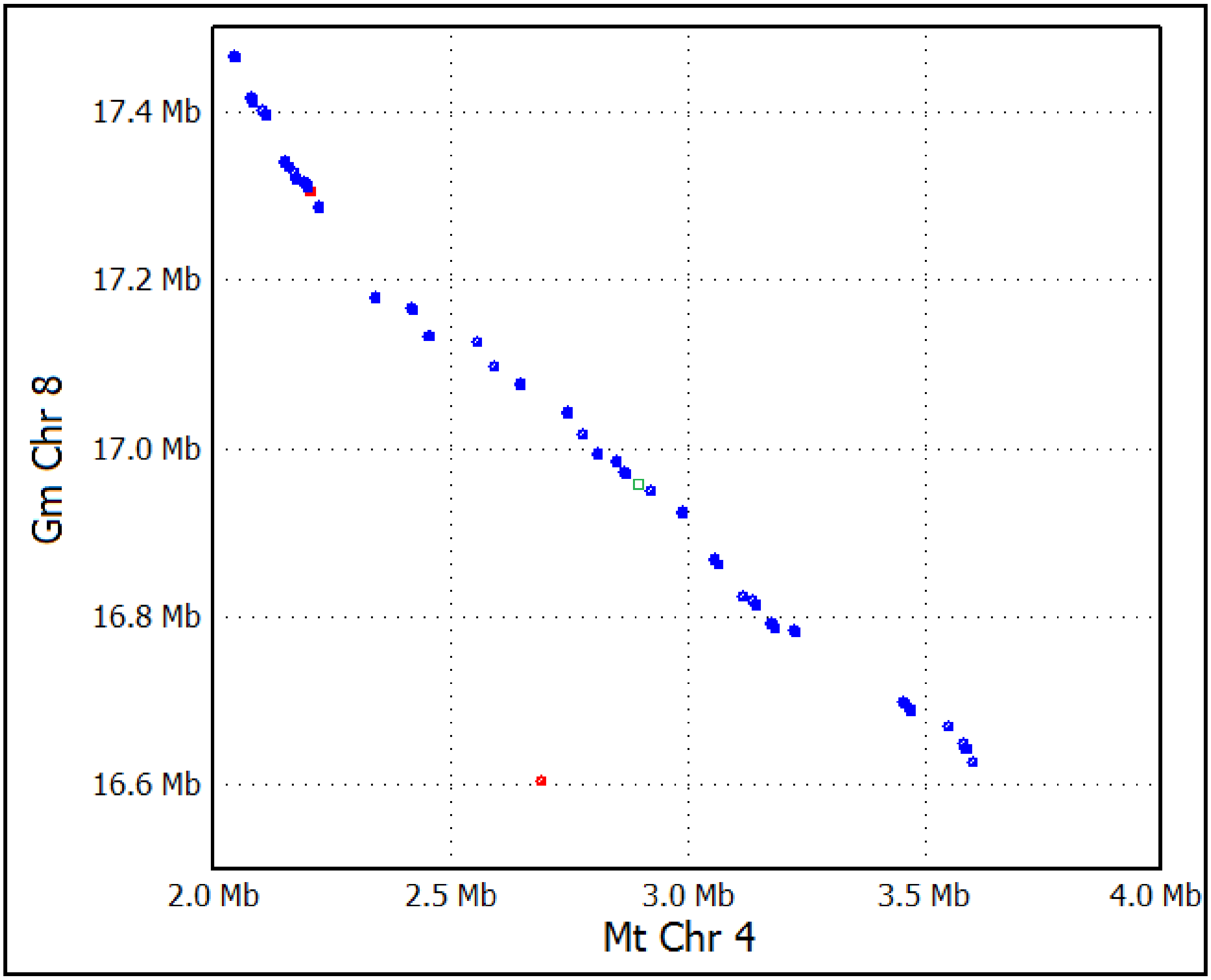

17.4 Mb
17.2 Mb
17.0 Mb
16.8 Mb
16.6 Mb
Gm Chr 8
2.0 Mb
2.5 Mb
3.0 Mb
3.5 Mb
4.0 Mb
Mt Chr 4

B.

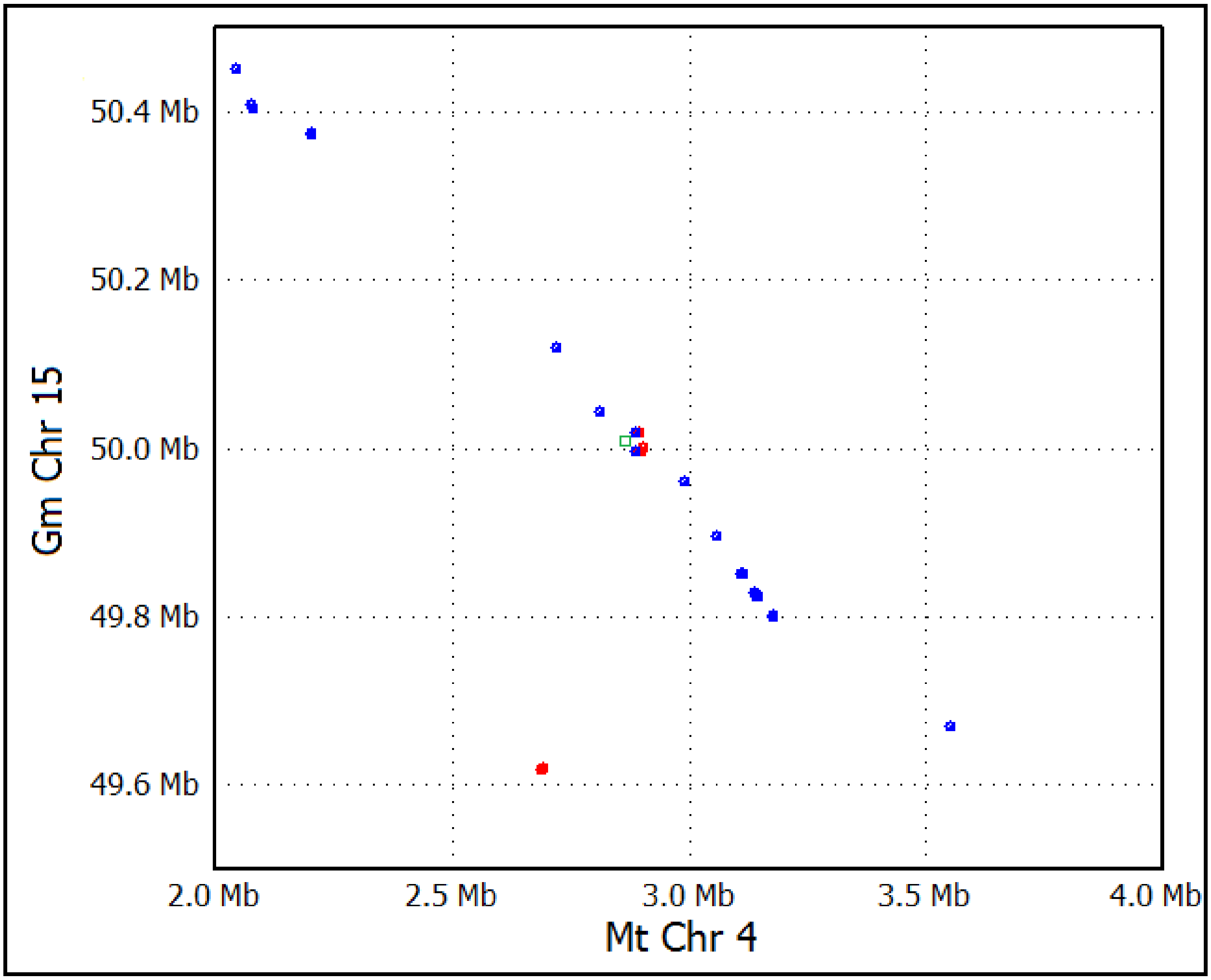

50.4 Mb
50.2 Mb
50.0 Mb
49.8 Mb
49.6 Mb
Gm Chr 15
2.0 Mb
2.5 Mb
3.0 Mb
3.5 Mb
4.0 Mb
Mt Chr 4

C.

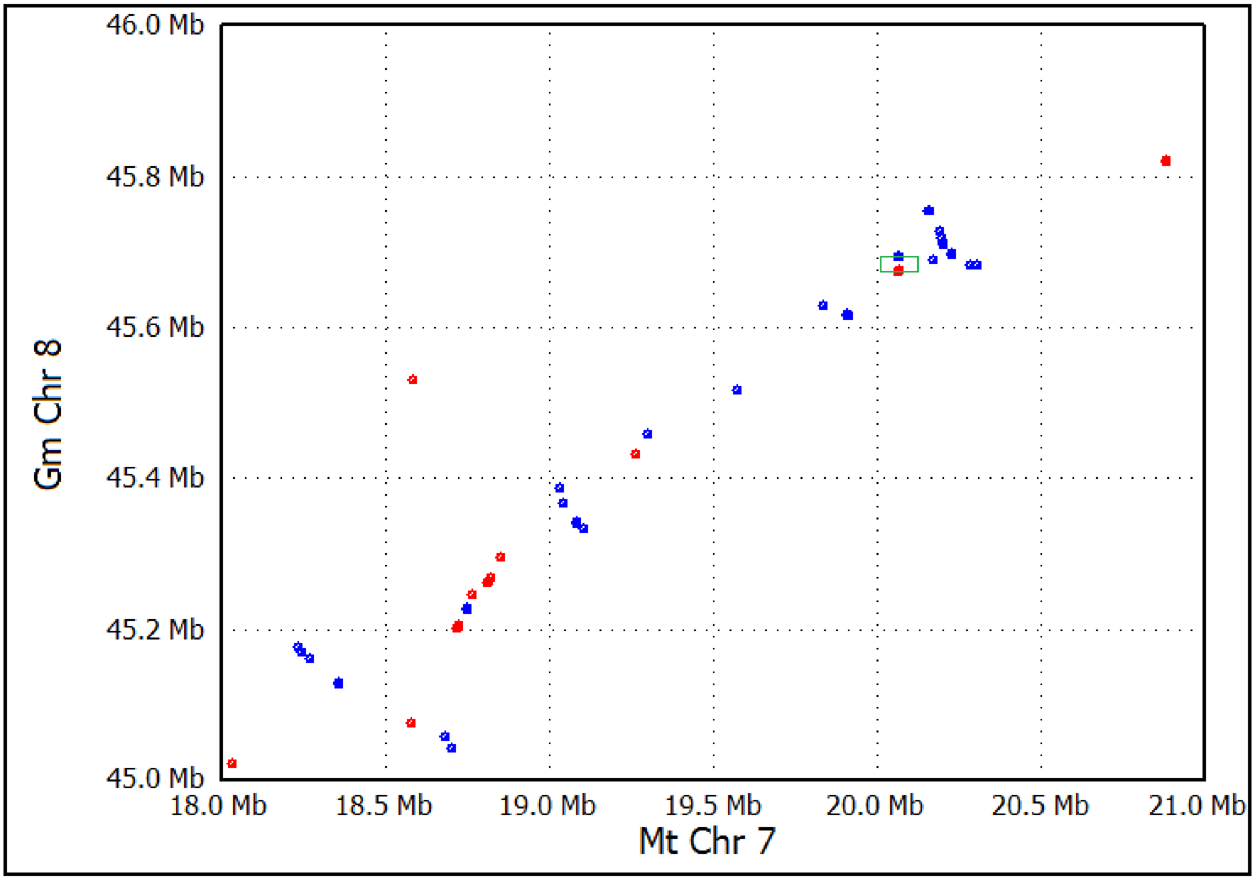
46.0 Mb
45.8 Mb
45.6 Mb
45.4 Mb
45.2 Mb
45.0 Mb
Gm Chr 8
18.0 Mb
18.5 Mb
19.0 Mb
19.5 Mb
20.0 Mb
20.5 Mb
21.0 Mb
Mt Chr 7

D.

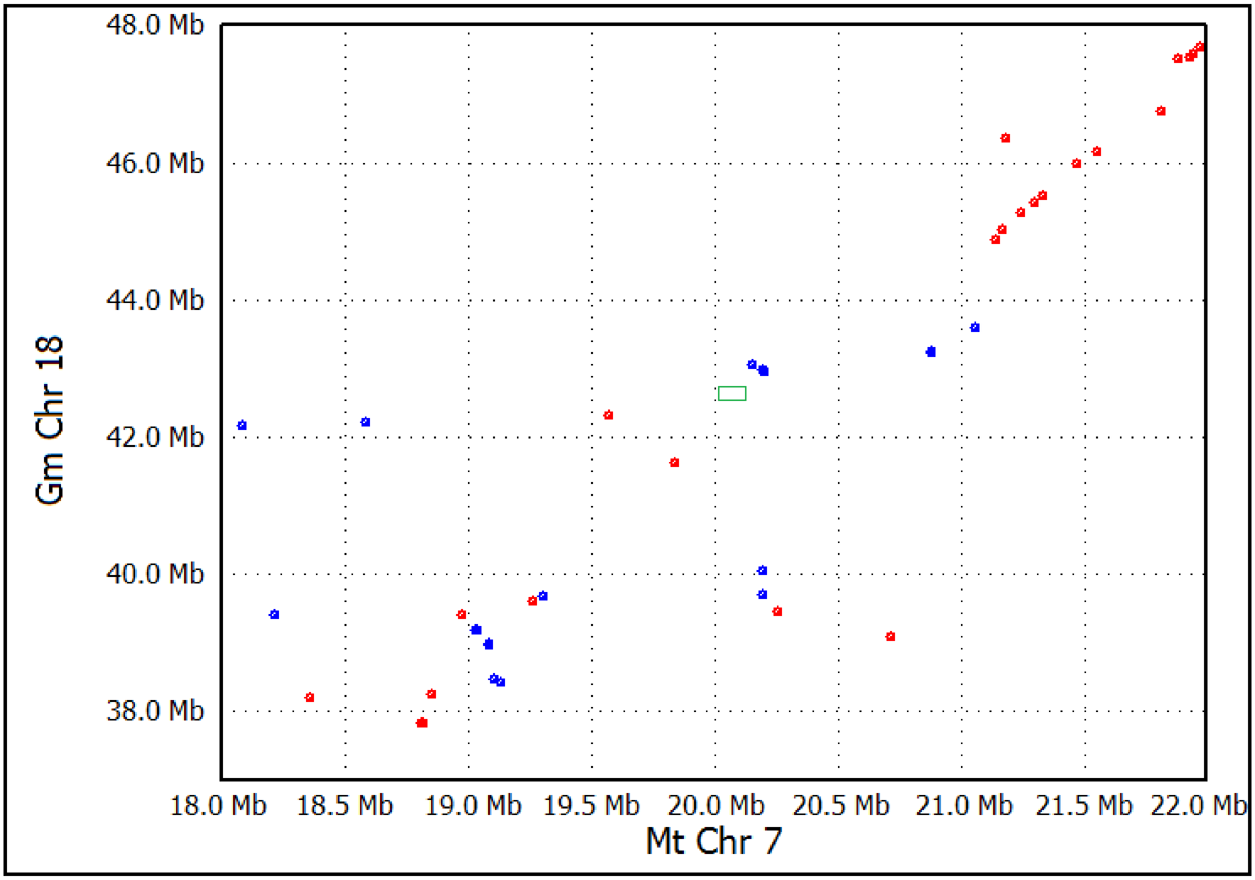

Figure S4 Dotplot comparisons between *M. truncatula* and *G. max* in regions surrounding *M. truncatula* LPs. Panels show syntenic regions of *G. max* chromosomes 8 a) and 15 b) compared against a 2 Mb region in A17 chromosome 4, corresponding to 0.5% of the total *M. truncatula* genome. Syntenic regions of *G. max* chromosomes 8 c) and 18 d) are compared against a 3-4 Mb region of A17 chromosome 7. Red dots indicate regions of sequence similarity with a forward orientation, while blue dots indicate reverse matches. The LP region in *M. truncatula* is shown with green rectangles.
