# Supplementary material for: Genomic Characterization of the LEED..PEEDs, a Gene Family Unique to the Medicago Lineage
Source: G3 (Bethesda). 2014 Aug 25;4(10):2003–12. doi: 10.1534/g3.114.011874 (PMC4199706; doi:10.1534/g3.114.011874)
Supplement: Supporting Information [file supp_g3.114.011874_FigureS5.pdf]

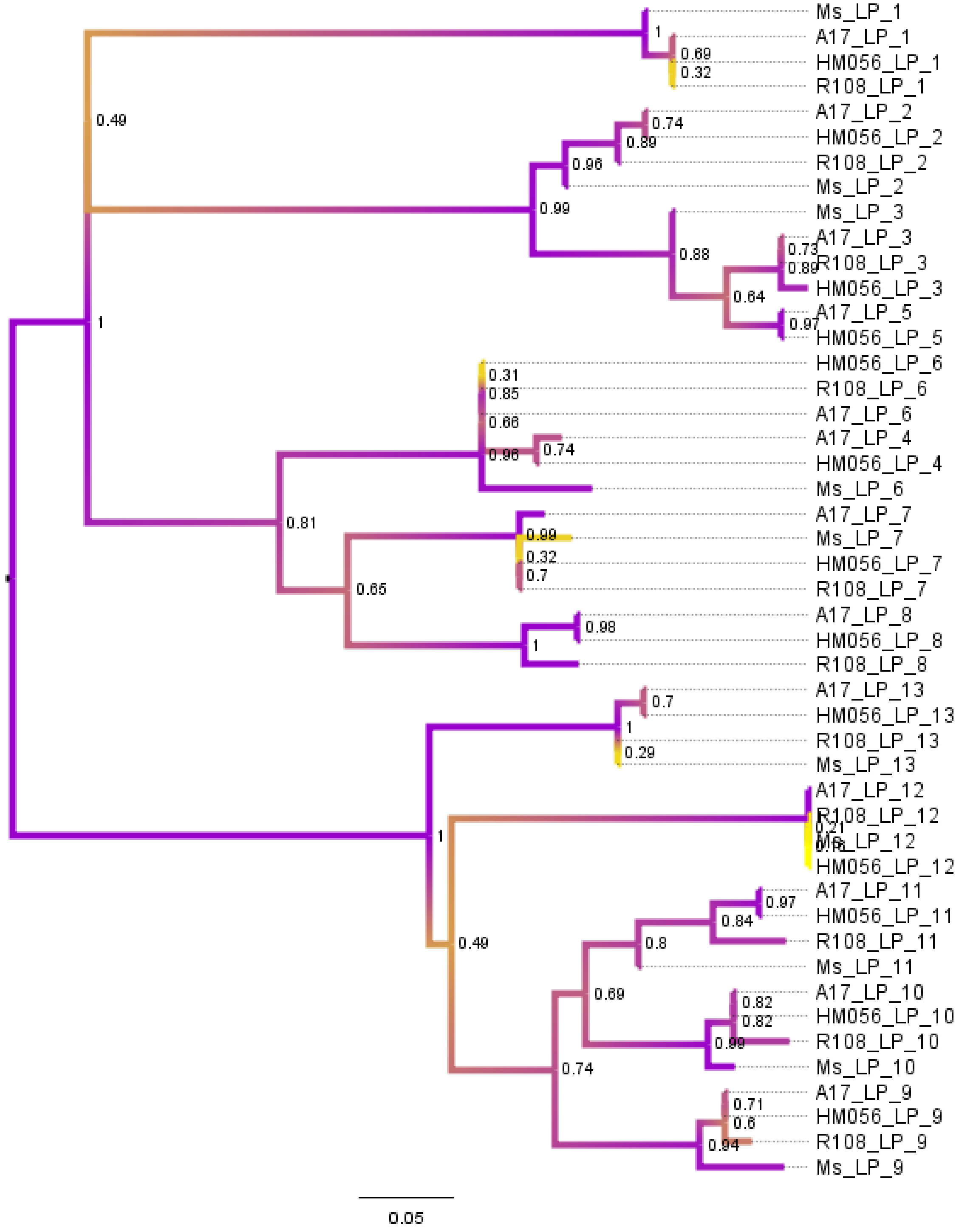

Figure S5 Phylogenetic tree of A17, HM056, R108 and *M. sativa* LP nucleotide sequences. The tree was generated through Bayesian Inference and visualized with FigTree software. Posterior probability values of the clades are indicated at the nodes.
